# Temporal Evolution of the Impact of CKD on COVID-19 Outcomes: A 5-Year Nationwide Analysis

**DOI:** 10.34067/KID.0000001133

**Published:** 2026-01-23

**Authors:** José D. González-Barajas, Manuel L. Prieto-Magallanes, Carolina Romo-Alvarez, Bladimir Díaz-Villavicencio, Violeta A. Camarena-Arteaga, Ana M. López-Yáñez, Judith C. De Arcos-Jiménez, Jaime Briseno-Ramirez

**Affiliations:** 1 Hospital Civil de Oriente, Tónala, Mexico; 2 Centro Universitario de Tlajomulco, Universidad de Guadalajara, Tlajomulco de Zúñiga, Mexico

**Keywords:** CKD, COVID-19, ESKD, epidemiology and outcomes, mortality, outcomes, clinical science

## Abstract

**Key Points:**

- CKD was associated with higher odds of coronavirus disease 2019 death and hospitalization across all phases in Mexico.
- Patients with CKD had lower intensive care unit/invasive mechanical ventilation use but higher in-intensive care unit fatality, especially at 18–64 years, which may reflect selection effects and triage decisions.
- Associations attenuated in 2022 but resurged by 2024; findings remained after municipal socioeconomic status adjustment.

**Background:**

CKD is among the strongest predictors of adverse coronavirus disease 2019 outcomes, yet how CKD-associated risk evolved across prevaccination, roll-out, mass-vaccination, and postpandemic phases remains incompletely defined in Latin America.

**Methods:**

We performed a nationwide retrospective analysis of Mexico's historical surveillance open datasets, including all laboratory-confirmed cases from January 1, 2020, to June 30, 2025. The primary outcome was death among recorded cases (case fatality); secondary outcomes were hospitalization, intensive care unit (ICU) admission, and invasive mechanical ventilation (IMV). Multivariable logistic models were fit overall, by age strata and year/pandemic phase, adjusting for demographics, comorbidities, and symptom-to-care interval; temporal patterns were summarized with locally estimated scatterplot smoothing and segmented interrupted time series analysis. Sensitivity analyses included models restricted to ICU admissions and models additionally adjusted for municipal socioeconomic context.

**Results:**

Among 7,359,354 cases, 70,144 (1.0%) had CKD; crude case fatality was 33% in CKD versus 4% without. In adjusted models, CKD was associated with higher case fatality (adjusted odds ratio [aOR], 1.20; 95% confidence interval [CI], 1.19 to 1.20) and hospitalization (aOR, 1.35; 95% CI, 1.35 to 1.35), but not with ICU admission (aOR, 0.98) or IMV (0.99). In the ICU-restricted cohort, CKD independently increased in-ICU case fatality (aOR, 1.38; 95% CI, 1.25 to 1.51), the strongest at age 18–64 years (aOR, 1.52) and smaller at ≥65 years (aOR, 1.19). Year-specific models showed attenuation during mass vaccination (2022 aOR, 1.13), followed by a resurgence in 2024 (aOR, 1.90). Locally estimated scatterplot smoothing/interrupted time series revealed delayed and incomplete postvaccination benefit for CKD with trend acceleration in 2023. The findings were robust after adding municipal socioeconomic status indicators to adult models.

**Conclusions:**

Across 5 years of nationwide surveillance, CKD remained a dominant, independent correlate of coronavirus disease 2019 case fatality and hospitalization, with risk resurging in 2024. Lower ICU/IMV use yet higher in-ICU case fatality among patients with CKD may reflect selection effects and underscore the need for rapid escalation pathways.

## Visual Abstract

**Figure:**
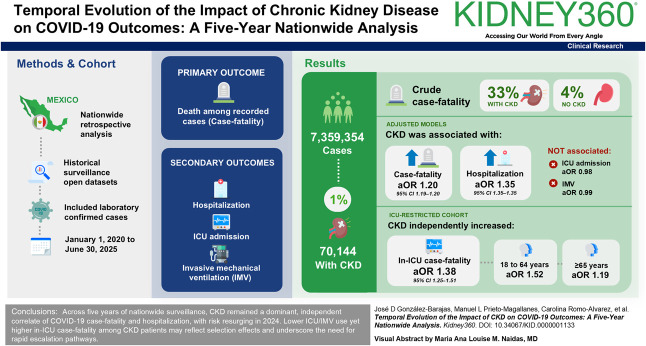

## Introduction

The coronavirus disease 2019 (COVID-19) pandemic has caused an estimated 14.8–18.2 million excess deaths worldwide, far exceeding official counts and reflecting both direct and indirect effects.^1–3^ Age, sex, and comorbidities are key risk stratifiers, strongly associated with severe outcomes and mortality in COVID-19.^4–7^ Common conditions such as diabetes, hypertension, and cardiovascular disease consistently increase the risk of intensive care unit (ICU) admission, mechanical ventilation, and death, independent of age, as shown in large cohorts and meta-analyses.^7–12^

CKD is globally prevalent and often coexists with those same conditions, amplifying overall vulnerability.^13^ CKD is now recognized as an independent risk factor for severe COVID-19–hospitalization, ICU admission, and death.^13–16^ The risk is especially high in advanced CKD (stages 4–5), dialysis, and kidney transplant, with adjusted hazard ratios for death of approximately 2.5–3.7—surpassing those for diabetes or chronic heart disease.^17,18^ Risk increases progressively with declining GFR, and even mild-moderate reductions confer greater severity and mortality.^19^

CKD entails chronic immune dysfunction, contributing to heightened infection risk and reduced vaccine responses.^20^ It also increases the likelihood of AKI during COVID-19, a strong predictor of mortality.^20,21^ Molecular studies suggest that CKD alters the expression of host genes (*e.g*., scavenger receptor class F [family of genes]) involved in severe acute respiratory syndrome coronavirus 2 entry and replication, although mechanisms remain incompletely understood.^20^

Despite its importance, CKD has been underrepresented in COVID-19 research—particularly in early reports and clinical trials—where it was frequently not specified as a risk factor or was undersampled.^15^ Under-recognition likely reflects overlap with other comorbidities, difficulties identifying CKD in administrative data, and routine exclusion of advanced CKD and transplant recipients from interventional studies.^16,17^

Given these gaps, we aimed to quantify how CKD-associated risk evolved across different pandemic phases and age groups. Using nationwide surveillance data and advanced time series methods, we examined outcomes across vaccine rollout periods. By adjusting for key demographic and clinical confounders, we sought to estimate changes over time and inform public health policies and clinical strategies to protect people living with CKD.

## Methods

### Data Source, Case Definition, and Study Population

We conducted a retrospective analysis of Mexico's nationwide open surveillance files from the General Directorate of Epidemiology (DGE; *Datos Abiertos – Bases Históricas*).^22^ Historical files updated as of July 1, 2025, were used. We restricted the cohort to laboratory-confirmed COVID-19 cases from January 1, 2020, to June 30, 2025.^22^ The DGE site provides the datasets and the accompanying data dictionary defining variable names and code lists.^22,23^ Because the Sistema de Vigilancia Epidemiológica de Enfermedades Respiratorias (National Respiratory Surveillance System) evolved over time, we harmonized case confirmation across file vintages using the fields and catalogs specified in the DGE data dictionary.^22,23^ Laboratory confirmation was derived from the test result and final case classification fields in each vintage (PCR or antigen positive with a confirmed classification). We included all deidentified person-level records meeting this definition and retained the earliest complete entry when duplicates shared the same person key.

### Variables and Coding

We extracted demographics, time from symptom onset to medical care, comorbidities, and severity indicators using the DGE data dictionary.^23^ Comorbidities are binary-coded. CKD is recorded as a single binary field indicating a preexisting diagnosis; CKD stage (*e.g*., eGFR), dialysis status, and kidney transplantation are not captured. Accordingly, all patients coded as having CKD were classified in the CKD group for our analyses, and we could not distinguish non–dialysis-dependent CKD from dialysis or transplant recipients. To facilitate interpretation across the life course, we analyzed prespecified age strata (0–4, 5–17, 18–64, and ≥65 years).

### Outcomes

The primary outcome was death among recorded cases (case fatality), defined by a valid death date in the vital status field of the national surveillance database. Because place of death is not captured, deaths cannot be classified as in-hospital or out-of-hospital. Secondary outcomes were hospital admission (inpatient versus ambulatory), ICU admission, and invasive mechanical ventilation (IMV), as defined by the severity indicators in the DGE open-data dictionary.^23^

### Data Quality Controls and Handling of Missingness

We excluded records missing age or sex and, for each outcome, omitted records with missing outcome data. Implausible ages (<0 or >120 years) were removed. For comorbidities and severity indicators with yes/no/unknown catalogs, yes was coded as present, no as absent, and unknown treated as missing in primary analyses. These procedures follow the structure and catalogs of the official open data dictionary.^23^

### Statistical Analysis

Crude rates were calculated as the proportion of patients experiencing each outcome, stratified by CKD status and age group (0–4, 5–17, 18–64, and ≥65 years). Adjusted probabilities were estimated using multivariable logistic regression, controlling for age, sex, diabetes, chronic obstructive pulmonary disease (COPD), asthma, immunosuppression, hypertension, cardiovascular disease, obesity, smoking status, other comorbidities, and time from symptom onset to the first medical encounter. We also fit multivariable logistic regression models for each outcome within each age stratum and calendar year to estimate adjusted odds ratios (aORs) for CKD and other covariates while controlling for potential confounders. Model performance metrics were computed to assess discrimination and classification performance (*e.g*., area under the curve for discrimination; accuracy, sensitivity, and specificity for classification).

We linked each case to municipality-level socioeconomic indicators *via* state—municipality residence codes and included these variables in multivariable models. Specifically, we merged (*1*) the social lag index (SLI; Consejo Nacional de Evaluación de la Política de Desarrollo Social [National Council for the Evaluation of Social Development Policy] 2020),^24^ which ranks municipalities by deprivations in education, health services, basic household services, housing quality/space, and assets and (*2*) the Marginalization Index (Consejo Nacional de Población [National Population Council] 2020),^25^ derived from census-based indicators of structural exclusion. We defined a binary low-socioeconomic status (SES) exposure equal to 1 if a municipality was classified as high or very high on either index and 0 otherwise. As a continuous proxy for access to care, we added the municipal share of residents without social security coverage (Instituto Nacional de Estadística y Geografía [National Institute of Statistics and Geography] 2020), scaled per 10%-point increase. SES variables were appended to person-level records by municipality of residence and entered as covariates in outcome-specific logistic regression models with demographics and comorbidities to estimate associations independent of individual risk factors.

To evaluate temporal dynamics in case fatality, we smoothed weekly case fatality proportions using locally estimated scatterplot smoothing (LOESS) and visualized trends for patients with and without CKD. We then fit interrupted time series (ITS) segmented linear regression models to estimate changes in the weekly proportion of COVID-19–related deaths in the overall cohort and the CKD subgroup. The study period comprised four segments: prevaccination (2020), early vaccination (2021), mass vaccination (2022), and postpandemic (2023–2025). For each group, the segmented model included an intercept (baseline level), a presegment trend, and period-specific level and slope changes at the start of each segment. Level changes capture immediate shifts at each cutpoint, whereas slope changes represent modifications in the weekly trajectory within each segment.

Analyses were conducted in R 4.3.2/RStudio on a high-performance computing cluster. Data wrangling (dplyr, tidyr, data.table), modeling (biglm, speedglm, nlme), evaluation (pROC, caret), and visualization (ggplot2, patchwork) were performed in R, with results summarized using broom and gtsummary. Geospatial processing and text normalization were performed in Python 3.12 with pandas, GeoPandas, and matplotlib.

This nationwide retrospective cohort study was analyzed and reported in accordance with the Strengthening the Reporting of Observational Studies in Epidemiology (STROBE) guidelines^26^; a completed STROBE checklist indicating where each item is addressed is provided in Supplemental Table 1.

### Ethical Statements

This study used fully anonymized, publicly available surveillance datasets from the Mexican Ministry of Health. According to national regulations, ethical approval and informed consent were not required. The study complies with the principles of the Declaration of Helsinki.

## Results

**Figure 1:**
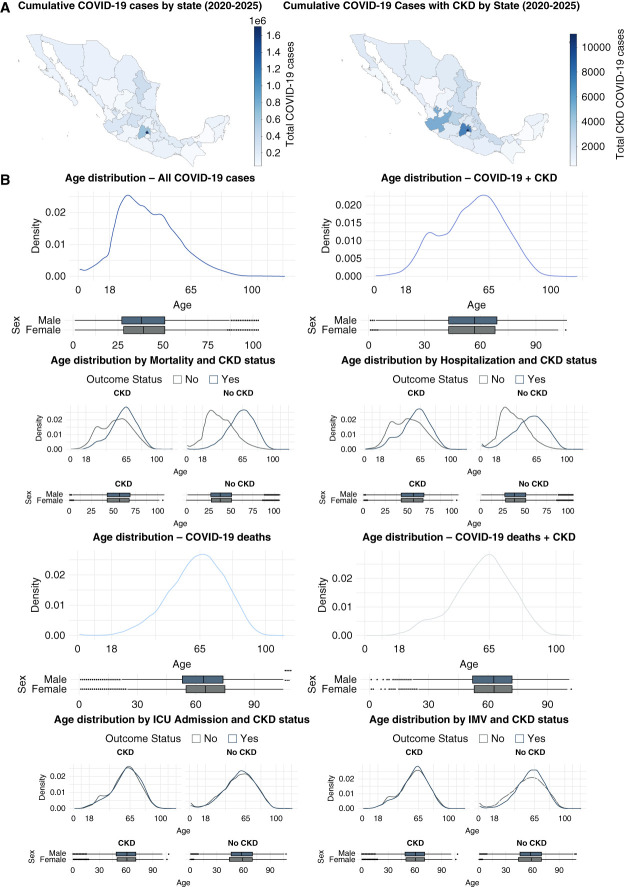
**Geographic and demographic characterization of COVID-19 cases in Mexico (2020–2025).** (A) Geographic distribution of cumulative laboratory-confirmed COVID-19 cases by state (left) and cumulative COVID-19 cases in the CKD subgroup by state (right). (B) Demographic analysis including age density distributions and sex-stratified boxplots for the overall cohort and the CKD subgroup. Sub-panels show age distributions stratified by clinical outcomes (mortality, hospitalization, ICU admission, and invasive mechanical ventilation) comparing patients with and without CKD. COVID-19, coronavirus disease 2019; ICU, intensive care unit; IMV, invasive mechanical ventilation.

From January 2020 to June 2025, 7,359,354 laboratory-confirmed COVID-19 cases were reported (46% male, 54% female; median age 38 years [interquartile range, 28–51]). Of these, 9.7% required hospitalization, 0.7% were admitted to intensive care, 1% underwent IMV, and 4% died. CKD was present in 70,144 patients (1%), with a case fatality rate of 33% versus 4% in those without CKD (case fatality ratio 8.25). Patients with CKD were older and had a higher burden of cardiometabolic comorbidities—particularly diabetes, hypertension, cardiovascular disease, and obesity—than those without CKD. Baseline sociodemographic characteristics, comorbidities, and outcomes stratified by CKD status are shown in Table 1, whereas sex-stratified characteristics are presented in Supplemental Table 2, and age, sex, and geographic distributions appear in Figure 1.

**Table 1:** Sociodemographic profile, comorbidities, and clinical outcomes of patients with laboratory-confirmed coronavirus disease 2019 stratified by CKD status (January 2020–June 2025)

Variable	*No.*	Overall (*N*=7,359,354)	CKD Status	
			Without CKD (*n*=7,289,210)	With CKD (*n*=70,144)
**Demographics**				
Age, yr, median (Q1–Q3)	7,359,354	38 (28–51)	38 (28–51)	57 (43–68)
Age group, yr, *No.* (%)	7,359,354			
*0–4*		77,088 (1.0)	76,987 (1.1)	101 (0.1)
*5–17*		392,794 (5.3)	392,038 (5.4)	756 (1.1)
*18–64*		6,238,339 (85)	6,192,496 (85)	45,843 (65)
*65+*		651,133 (8.8)	627,689 (8.6)	23,444 (33)
Sex, *No.* (%)	7,359,354			
*Female*		3,957,261 (54)	3,925,312 (54)	31,949 (46)
*Male*		3,402,093 (46)	3,363,898 (46)	38,195 (54)
Indigenous status, *No.* (%)	7,062,904	52,530 (0.7)	51,799 (0.7)	731 (1.1)
Migrant status, *No.* (%)	7,359,354	4586 (<0.1)	4560 (<0.1)	26 (<0.1)
**Comorbidities and clinical characteristics, No. (%)**				
Diabetes	7,337,963	647,852 (8.8)	611,457 (8.4)	36,395 (52)
COPD	7,338,990	51,160 (0.7)	47,214 (0.6)	3946 (5.6)
Asthma	7,339,198	140,837 (1.9)	138,805 (1.9)	2032 (2.9)
Immunosuppression	7,338,991	43,598 (0.6)	38,176 (0.5)	5422 (7.7)
Hypertension	7,338,894	884,751 (12)	838,217 (12)	46,534 (66)
Cardiovascular disease	7,338,980	74,946 (1.0)	67,287 (0.9)	7659 (11)
Obesity	7,339,968	714,144 (9.7)	701,887 (9.7)	12,257 (17)
Smoking	7,338,611	391,798 (5.3)	385,438 (5.3)	6360 (9.1)
Pregnancy	3,927,696	65,715 (1.7)	65,588 (1.7)	127 (0.4)
Other comorbidities	7,298,305	115,832 (1.6)	110,987 (1.5)	4845 (7.0)
Days from symptoms onset to care, median (Q1–Q3)	7,359,354	2 (1–4)	2 (1–4)	3 (1–5)
**Outcomes, No. (%)**				
Case fatality	7,359,354	311,906 (4.2)	288,994 (4.0)	22,912 (33)
Hospitalization	7,359,354	710,285 (9.7)	669,075 (9.2)	41,210 (59)
ICU admission	704,582	52,639 (0.7)	50,323 (7.6)	2316 (5.6)
IMV	704,581	80,251 (1.1)	75,709 (11)	4542 (11)

COPD, chronic obstructive pulmonary disease; ICU, intensive care unit; IMV, invasive mechanical ventilation.

The median age at death was 65 years (interquartile range, 54–74), versus 37 years (27–50) in survivors. Nonsurvivors had longer delays to care (median 5 versus 2 days). Comorbidities were far more frequent in deaths, notably diabetes (37% versus 8%), COPD (5% versus 0.5%), hypertension (45% versus 11%), cardiovascular disease (5% versus 0.8%), obesity (21% versus 9%), and CKD (7% versus 0.7%). Immunosuppression, smoking, and indigenous status were also more common. Table 2 summarizes descriptive and adjusted results for case fatality.

**Table 2:** Descriptive characteristics and adjusted odds ratios for case fatality and hospitalization in patients with laboratory-confirmed coronavirus disease 2019

Variable	Total	Death		Multivariate Regression Analysis				Hospitalization		Multivariate Regression Analysis			
	*N*=7,359,354	Absent	Present	aOR	95% CI Lower	95% CI Upper	*P* Value	Absent	Present	aOR	95% CI Lower	95% CI Upper	*P* Value
		*n*=7,047,448	*n*=311,906					*n*=6,649,069	*n*=710,285				
**Sex, *No.* (%)**													
Male	3,402,093 (46)	3,210,848 (46)	191,245 (61)	1.024	1.024	1.025	<0.001	2,995,792 (45)	406,301 (57)	1.041	1.04	1.041	<0.001
Female	3,957,261 (54)	3,836,600 (54)	120,661 (39)					3,653,277 (55)	303,984 (43)	—	—	—	—
Age, median (Q1–Q3)	38.0 (28.0–51.0)	37.0 (27.0–50.0)	65.0 (54.0–74.0)	1.002	1.002	1.002	<0.001	37.0 (27.0–49.0)	59.0 (45.0–70.0)	1.004	1.004	1.004	<0.001
CKD, *No.* (%)	70,144 (1)	47,232 (0.7)	22,912 (7)	1.195	1.193	1.197	<0.001	28,934 (0.4)	41,210 (6)	1.349	1.346	1.351	<0.001
Diabetes, *No.* (%)	647,852 (9)	532,921 (8)	114,931 (37)	1.061	1.061	1.062	<0.001	429,911 (7)	217,941 (31)	1.125	1.124	1.125	<0.001
COPD, *No.* (%)	51,160 (0.7)	36,970 (0.5)	14,190 (5)	1.112	1.11	1.114	<0.001	25,101 (0.4)	26,059 (4)	1.211	1.208	1.214	<0.001
Asthma, *No.* (%)	140,837 (2)	135,348 (2)	5489 (2)	0.98	0.98	0.99	<0.001	126,461 (2)	14,376 (2)	0.99	0.988	0.991	<0.001
Immunosuppression, *No.* (%)	43,598 (0.6)	36,085 (0.5)	7513 (2)	1.049	1.047	1.051	<0.001	26,600 (0.4)	16,998 (2)	1.161	1.158	1.164	<0.001
Hypertension, *No.* (%)	884,751 (12)	745,575 (11)	139,176 (45)	1.035	1.035	1.036	<0.001	626,488 (9)	258,263 (36)	1.06	1.06	1.061	<0.001
Cardiovascular disease, *No.* (%)	74,946 (1)	58,674 (0.8)	16,272 (5)	1.054	1.053	1.056	<0.001	43,336 (0.7)	31,610 (5)	1.123	1.12	1.125	<0.001
Obesity, *No.* (%)	714,144 (10)	649,322 (9)	64,822 (21)	1.015	1.015	1.016	<0.001	581,780 (9)	132,364 (19)	1.031	1.03	1.031	<0.001
Smoking, *No.* (%)	391,798 (5)	368,454 (5)	23,344 (8)	0.997	0.997	0.998	<0.001	341,931 (5)	49,867 (7)	0.995	0.994	0.996	<0.001
Pregnancy, *No.* (%)	65,715 (2)	65,330 (2)	385 (0.3)	—	—	—	—	56,293 (0.9)	9422 (1)	—	—	—	—
Other comorbidity, *No.* (%)	115,832 (2)	99,550 (1)	16,282 (5)	1.054	1.053	1.056	<0.001	81,342 (1)	34,490 (5)	1.128	1.127	1.13	<0.001
Indigenous status, *No.* (%)	52,530 (0.7)	47,713 (0.7)	4817 (2)	1.027	1.025	1.029	<0.001	42,231 (0.7)	10,299 (1)	1.061	1.059	1.064	<0.001
Migrant status, *No.* (%)	4586 (0.06)	4455 (0.06)	131 (0.04)	—	—	—	—	4031 (0.06)	555 (0.08)	—	—	—	—
Time from symptoms to attention, median (Q1–Q3)	2.0 (1.0–4.0)	2.0 (1.0–4.0)	5.0 (2.0–7.0)	1.008	1.008	1.008	<0.001	2.0 (1.0–4.0)	4.0 (2.0–7.0)	1.018	1.018	1.018	<0.001

Model performance metrics for case fatality: AUC 0.90, accuracy 0.95.

Model performance metrics for hospitalization: AUC 0.84, accuracy 0.90. aOR, adjusted odds ratio; AUC, area under the curve; CI, confidence interval; COPD, chronic obstructive pulmonary disease.

In multivariable models, CKD was the strongest independent predictor of death (aOR, 1.195; 95% confidence interval [CI], 1.193 to 1.197) and was associated with 35% higher odds of hospitalization (aOR, 1.349; 95% CI, 1.346 to 1.351), both *P* < 0.001 (Table 2).

CKD was not associated with increased ICU admission (aOR, 0.981) or IMV (aOR, 0.989). Instead, male sex, obesity, and immunosuppression were associated with both outcomes; age and diabetes had minimal effect. Full details appear in Supplemental Table 3.

**Figure 2:**
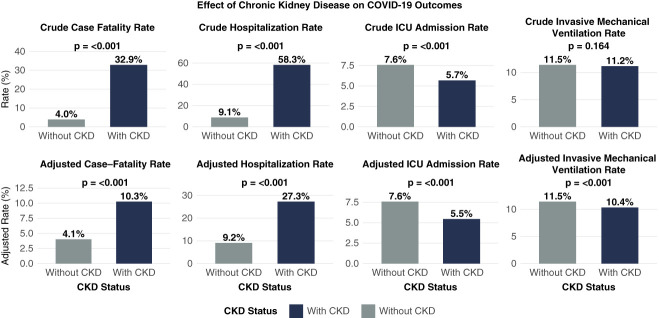
Crude and adjusted rates of COVID-19 outcomes by CKD status among laboratory-confirmed cases.

Patients with CKD had substantially higher crude case fatality (33% versus 4.0%, *P* < 0.001) and hospitalization rates (58% versus 9%, *P* < 0.001) compared with those without CKD. After multivariable adjustment, these differences remained significant: Adjusted case fatality was 10.3% in CKD versus 4.1% without CKD (*P* < 0.001), and adjusted hospitalization was 27.3% versus 9.2% (*P* < 0.001). ICU and IMV use were not higher among patients with CKD; ICU admission was lower in CKD in both crude (5.7% versus 7.6%) and adjusted analyses (5.5% versus 7.6%; both *P* < 0.001), and invasive ventilation rates were similar or slightly lower (crude 11.5% versus 11.2%; adjusted 10.4% versus 11.5%; both *P* < 0.001) as shown in Figure 2.

Age-stratified models identified CKD as a strong mortality predictor: aOR 2.02 in ≥65 years, 5.54 in 18–64 years, and 12.85 in 5–17 years. CKD also increased hospitalization risk, especially in 5–17 years (aOR up to 11.48). By contrast, CKD was not associated with higher ICU/IMV in adults and was associated with lower adjusted odds in some groups. In children, CKD increased odds of IMV (aOR, approximately 2–3.3). Full results are shown in Supplemental Tables 4–7.

**Figure 3:**
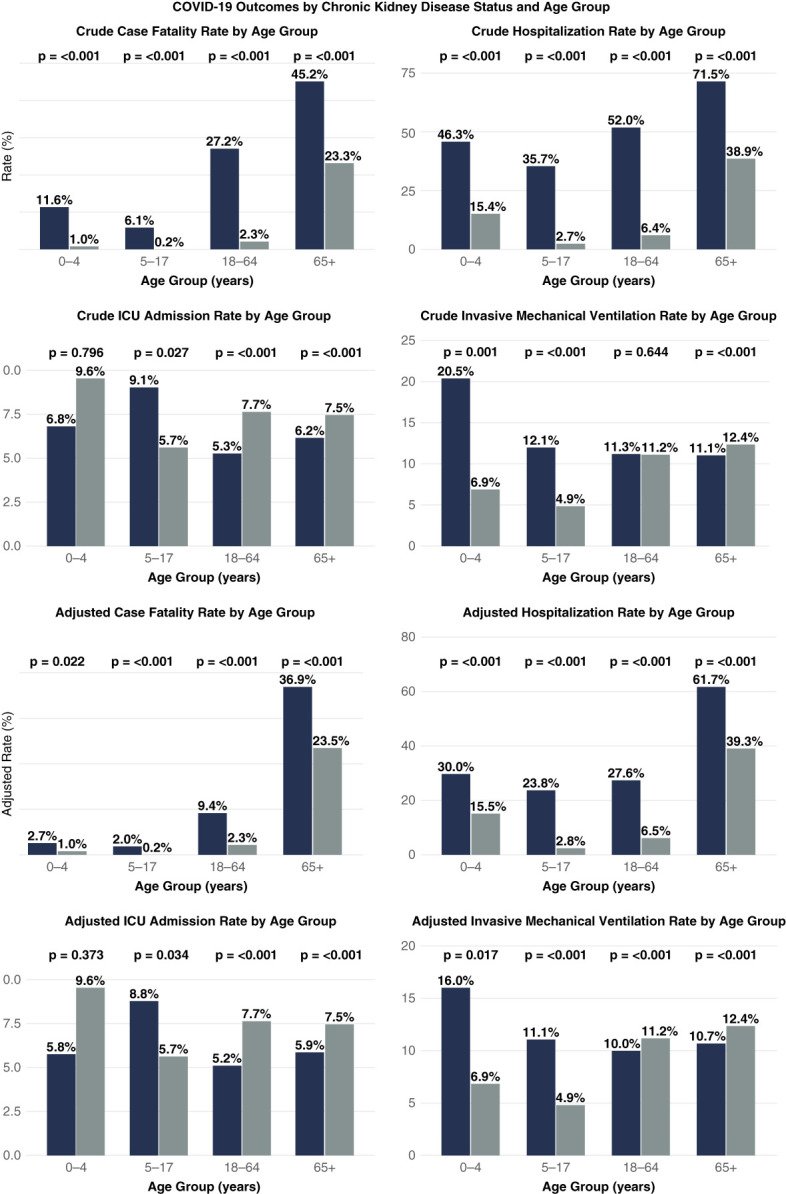
Age-stratified crude and adjusted rates of COVID-19 outcomes by CKD status among laboratory-confirmed cases.

CKD-related risk was consistent across age: in ≥65 years, fatality and hospitalization were higher in CKD (crude 45.2% versus 23.3%; 71.5% versus 38.9%; adjusted 36.9% versus 23.5%; 61.7% versus 39.3%). In 18–64 years, CKD similarly increased fatality (27.2% versus 2.3%) and hospitalization (52.0% versus 6.4%), with no ICU/IMV increase. In children, CKD conferred higher adjusted IMV (0–4 years: 16.0% versus 6.9%; 5–17 years: 11.1% versus 4.9%) (Figure 3).

In sensitivity analysis restricted to ICU admissions (*n*=52,639), CKD was associated with higher in-ICU case fatality (aOR, 1.38; 95% CI, 1.25 to 1.51; *P* < 0.001). Crude in-ICU fatality was 66.7% in CKD versus 56.4% in non-CKD. By age, the association was the strongest in adults age 18–64 years (aOR, 1.52; 95% CI, 1.34 to 1.73) and smaller but significant in ≥65 years (aOR, 1.19; 95% CI, 1.03 to 1.38); pediatric strata yielded imprecise, nonsignificant estimates because of very small CKD counts. See Supplemental Table 8 for details.

In multivariable grouped-binomial models adjusted for age, sex, and municipal SES indicators—(*1*) the municipal share without social security health coverage (Instituto Nacional de Estadística y Geografía [National Institute of Statistics and Geography] 2020, scaled per 10%-point increase) and (*2*) a low SES indicator for municipalities with high/very-high social lag and/or marginalization (Consejo Nacional de Evaluación de la Política de Desarrollo Social [National Council for the Evaluation of Social Development Policy] SLI 2020/Consejo Nacional de Población [National Population Council] 2020)—CKD remained a strong predictor of worse outcomes. For case fatality: CKD aOR=5.69 (95% CI, 5.58 to 5.80); age per year aOR=1.086; male sex aOR=1.91; no social security coverage aOR=1.15; Low SES aOR=1.41. For hospitalization: CKD aOR=8.87 (8.72 to 9.03); age aOR=1.072; male sex aOR=1.68; no social security coverage aOR=1.19; Low SES aOR=1.72. Full adjusted estimates (including ICU and IMV) are shown in Table 3.

**Table 3:** Adjusted odds ratios for coronavirus disease 2019 outcomes in adults (≥18 years), controlling for CKD, age, sex, and municipal SES derived from CONEVAL social lag (IRS 2020) and CONAPO marginalization (2020), plus no social security health coverage (INEGI 2020)

Outcome (Cohort)	CKD, aOR (95% CI)	Age (per Year), aOR (95% CI)	Male, aOR (95% CI)	No Social Security Health Coverage, aOR (95% CI)^a^	Low SES, aOR (95% CI)^b^
Case fatality	5.690 (5.584 to 5.799)	1.086 (1.086 to 1.086)	1.914 (1.899 to 1.930)	1.152 (1.145 to 1.158)	1.410 (1.373 to 1.449)
Hospitalization	8.871 (8.716 to 9.028)	1.072 (1.072 to 1.072)	1.684 (1.674 to 1.693)	1.194 (1.189 to 1.198)	1.723 (1.690 to 1.757)
ICU (all recorded cases)	0.729 (0.698 to 0.761)	1.001 (1.000 to 1.001)	1.225 (1.202 to 1.248)	0.952 (0.940 to 0.963)	1.278 (1.212 to 1.348)
IMV (all recorded cases)	0.942 (0.912 to 0.972)	1.007 (1.007 to 1.007)	1.297 (1.278 to 1.318)	1.091 (1.080 to 1.102)	0.972 (0.926 to 1.021)^c^

All models adjusted for age, sex, and municipal SES (see notes). Outcome definitions follow the national surveillance datasets (General Directorate of Epidemiology; acronym from Spanish).

Models' performance. Case fatality: AUC 0.871, Youden *t*≈0.04 → Acc 0.767/Sens 0.828/Spec 0.764/PPV 0.142/NPV 0.990/F1 0.243; hospitalization: AUC 0.809, Youden *t*≈0.10 → Acc 0.752/Sens 0.726/Spec 0.755/PPV 0.247/NPV 0.961/F1 0.369; intensive care unit (all cases): AUC 0.537, Youden *t*≈0.078 → Acc 0.543/Sens 0.514/Spec 0.546/PPV 0.083/NPV 0.933/F1 0.143; invasive mechanical ventilation (all cases): AUC 0.552, Youden *t*≈0.116 → Acc 0.527/Sens 0.553/Spec 0.524/PPV 0.132/NPV 0.900/F1 0.213. aOR, adjusted odds ratio; AUC, area under the curve; CI, confidence interval; CONAPO, Consejo Nacional de Población (National Population Council); CONEVAL, Consejo Nacional de Evaluación de la Política de Desarrollo Social (National Council for the Evaluation of Social Development Policy); ICU, intensive care unit; IMV, invasive mechanical ventilation; INEGI, Instituto Nacional de Estadística y Geografía (National Institute of Statistics and Geography); IRS, Índice de Rezago Social (social lag index); NPV, negative predictive value; PPV, positive predictive value; SES, socioeconomic status.

a Municipal share of residents without social security health coverage (no social security health coverage), scaled in 10%-point increments (*i.e*., a 10%-point increase). Source: INEGI 2020 Right-to-Care/social security health coverage statistics (Censo 2020).

b Low SES: indicator=1 if the municipality has high/very-high social deprivation by either (*1*) CONEVAL 2020 social lag index or (*2*) CONAPO 2020 marginalization index degree; 0 otherwise. CONEVAL IRS aggregates education, health service access, basic dwelling services, housing quality/space, and household assets into a single index with ordered degrees; CONAPO marginalization summarizes multiple deprivation components and publishes degrees at municipal level.

c Wald tests for all coefficients are *P* < 0.001, except for low SES in invasive mechanical ventilation (*P* = 0.257), which is not statistically significant at *α*=0.05.

Year-specific multivariable models (2020–2025) show that CKD was consistently associated with higher case fatality, attenuating from the prevaccination and early vaccination years (2020 aOR, 1.22; 2021 aOR, 1.24) to 2022–2023 (aOR, 1.13 and 1.05), then surging in 2024 (aOR, 1.90) before becoming imprecise and nonsignificant in early 2025 (aOR, 1.29; *P* = 0.35). Across all years, other comorbidities—particularly COPD (aOR range, 1.04–1.29), immunosuppression (up to aOR 1.75 in 2024), diabetes (up to aOR, 1.54), and indigenous ethnicity (up to aOR 3.58 in 2025)—also showed significant, although variable, associations with case fatality. Full models for each year are available in Supplemental Table 9.

**Figure 4:**
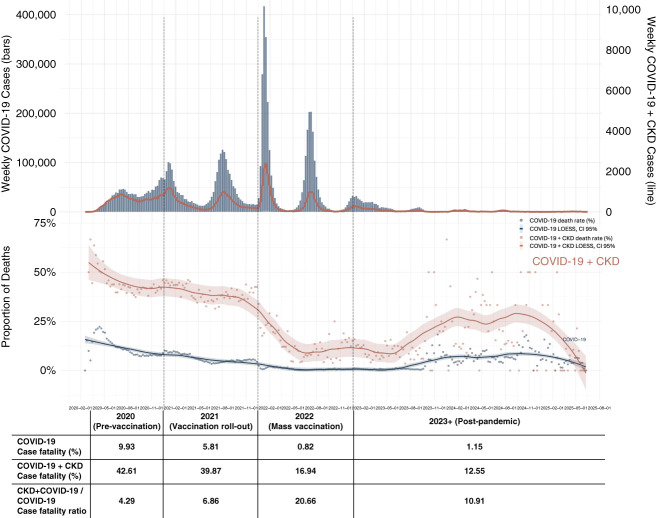
**Weekly COVID-19 case counts and death proportions by CKD status, with LOESS smoothing across pandemic phases.** Mexico's vaccination program began on December 24, 2020 (prevaccination phase in 2020), scaled through 2021 with adults covered in descending age bands and high-risk conditions (including CKD), and reached high adult coverage by October 29, 2021 (83% with ≥1 dose; 81% complete). Booster doses started in December 2021 (≥60 years; health care workers) and were extended to all adults in Q1-2022, marking the mass-vaccination period in 2022. Survey data indicate adult coverage around 86.6% (20–59 years) and 89.2% (≥60 years) by 2022, whereas booster uptake remained markedly lower than primary series coverage during 2022–2025. No public dataset reports vaccination coverage specifically among patients with CKD in Mexico; phases here are aligned to national roll-out milestones. See Supplemental Table 10 for yearly coverage data. CI, confidence interval; LOESS, locally estimated scatterplot smoothing.

Phase-stratified trends aligned with Mexico's vaccine roll-out show a sharp drop in overall case fatality from 10% (2020, prevaccination) to 6% (2021) and 0.82% (2022), followed by a modest rebound to approximately 1% from 2023 onward. By contrast, patients with CKD maintained substantially higher fatality—approximately 43% (2020), 40% (2021), 17% (2022), and approximately 13% thereafter—so the CKD-to-non-CKD case fatality ratio widened as population risk declined (≈4.3 in 2020 → 6.9 in 2021 → 20.7 in 2022) and remained >10 since 2023. Weekly LOESS curves in Figure 4 mirror these patterns, with persistent separation between CKD and non-CKD throughout all phases; for national adult vaccination coverage milestones that contextualize the roll-out phases, see Supplemental Table 10.^27–30^

Finally, ITS analysis using segmented regression of LOESS-smoothed weekly case fatality proportions showed persistently higher levels in CKD versus non-CKD at baseline (*β*_0_ 0.525 versus 0.157; both *P* < 0.001) and steeper initial declines in CKD (*β*_1_ −0.003 versus −0.002; both *P* < 0.001). Among all confirmed cases, significant step-ups and trend accelerations occurred at the 2022 and 2023 cut points (*β*_2_=0.038 and 0.131; *β*_3_≥0.001; all *P* ≤ 0.004). By contrast, CKD models showed no significant immediate level reductions at the 2021–2022 vaccination cut points (*β*_2_: 2021 *P* = 0.10; 2022 *P* = 0.43) and no additional downward trend change (*β*_3_: 2021 *P* = 0.25; 2022 *P* = 0.75), indicating attenuated short-term reductions in case fatality. Instead, CKD fatality declined gradually (baseline slope *β*_1_=−0.003, *P* < 0.001) and then exhibited a delayed upward inflection in 2023 (*β*_3_=0.003, *P* < 0.001). Full ITS analysis results are presented in Table 4.

**Table 4:** Interrupted time series analysis of coronavirus disease 2019 case fatality trends by CKD status and pandemic period

Parameter	COVID-19 Confirmed Patients				CKD+COVID-19 Confirmed Patients			
	*β*	95% CI Low	95% CI High	*P* Value	*β*	95% CI Low	95% CI High	*P* Value
*β*_0_ (constant)	0.157	0.148	0.166	<0.001	0.525	0.490	0.561	<0.001
*β*_1_ (time)	−0.002	−0.002	−0.001	<0.001	−0.003	−0.004	−0.002	<0.001
***β***_**2**_ **(level)**								
2021	0.007	−0.005	0.019	0.27	0.037	−0.008	0.081	0.1
2022	0.038	0.012	0.065	0.004	−0.041	−0.142	0.061	0.43
2023	0.131	0.089	0.173	<0.001	0.059	−0.105	0.223	0.48
***β***_**3**_ **trend**								
2021	0.001	0.000	0.001	<0.001	0.001	−0.001	0.003	0.25
2022	0.001	0.001	0.002	<0.001	0.000	−0.001	0.002	0.75
2023	0.002	0.002	0.003	<0.001	0.003	0.002	0.005	<0.001

CI, confidence interval; COVID-19, coronavirus disease 2019.

## Discussion

In this 5-year nationwide analysis of laboratory-confirmed COVID-19 in Mexico, CKD was consistently associated with worse outcomes. Across all phases, crude case fatality was approximately 33% in CKD versus approximately 4% without CKD (approximately 8:1). In multivariable models adjusted for age, sex, comorbidities, and symptom-to-care interval, CKD was associated with higher odds of death (aOR≈1.20) and hospitalization (aOR≈1.35); these differences persisted across stratified models (Figures 2 and 3).

Phase- and year-specific models showed significant CKD–mortality associations in 2020 (aOR, approximately 1.22) and 2021 (approximately 1.24), attenuation in 2022 (approximately 1.13), near-null in 2023 (approximately 1.05), and resurgence in 2024 (approximately 1.90); 2025 estimates were imprecise. LOESS and ITS models showed a delayed, incomplete postvaccination benefit in CKD, with later trend acceleration compared with the general population. This may reflect waning immunity, variant turnover, and suboptimal booster uptake.^31–33^

Our findings align with international evidence establishing CKD as a major risk factor for severe COVID-19.^14,17–19^ For example, CKD confers risks of ICU admission, IMV, and death that exceed those of diabetes and cardiovascular disease, with case fatality hazard ratios approximately 2.5–3.7 in advanced CKD/dialysis.^14,17–19^ Our observed eight-fold difference in crude case fatality is concordant with previous reports.^4,14,18,34^

Importantly, our study builds on previous work by showing elevated risk persisted into vaccine and postvaccine periods.^35,36^ Studies from high-income settings also report high postvaccine mortality among patients with CKD, with up to eight-fold fatality risk after breakthrough infection and attenuated vaccine responses.^35,36^

After adjusting for municipal socioeconomic status (SLI, Marginalization Index, and social security coverage), CKD remained strongly associated with death (aOR, 5.69) and hospitalization (aOR, 8.87). Deprivation and access barriers were independently linked to worse outcomes, suggesting that structural inequities amplify—but do not fully explain—the CKD effect.

Our study also contributes Latin American data, a region underrepresented in early pandemic research. The magnitude of CKD-associated risk in Mexico equals or exceeds other reports (*e.g*., Brazilian cohort, hazard ratio approximately 1.14).^37^ We observed 10%–20% higher adjusted odds of death in the postvaccine era and high fatality during surges (approximately 43.6% in 2020), comparable with dialysis cohorts in Latin America.^38^

Compared with published cohorts from high-income countries and Latin America, our CKD population shared similar risk factors—older age and high burden of diabetes, hypertension, and obesity—but showed distinct features. Although most international studies focus on hospitalized dialysis or transplant patients aged >70 years,^14,15,17,18,34,37^ our national surveillance cohort included both ambulatory and hospitalized cases and had a broader age distribution (median age 57 in CKD). Despite this, patients with CKD in Mexico had higher crude fatality (approximately 33%) than those reported in US, European, or Brazilian dialysis cohorts (typically 18%–28%),^15,18,37^ suggesting possible differences in access to ICU-level care, early interventions, or treatment resources. These contextual differences may partly explain why our relative effect sizes (aOR, approximately 1.2 for death) are lower than those from hospital-based studies (hazard ratios, 2.5–3.7),^15,17,18^ but the persistently high absolute mortality highlights the extreme vulnerability of patients with CKD in low-resource settings.

CKD was not consistently associated with higher ICU or IMV use. Some adult subgroups showed lower ICU/IMV use despite higher case fatality, which is consistent with the hypothesis that surge conditions and triage decisions, including a perceived limited benefit of intensive interventions in adults with CKD, may have contributed to this pattern. However, triage processes and treatment-limitation decisions were not captured in the surveillance database, so this explanation remains speculative and should be interpreted with caution. By contrast, pediatric CKD had higher odds of ventilatory support, suggesting different clinical practices in children. These findings are consistent with previous reports and underscore the role of resource allocation and clinical judgment in shaping outcomes for patients with CKD.^34,39^ To explore potential confounding by indication or selection bias, we performed a sensitivity analysis restricted to ICU patients, which showed that CKD remained independently associated with increased in-ICU mortality (overall aOR, 1.38; ages, 18–64: aOR, 1.52; ≥65: aOR, 1.19). These results suggest that lower aggregate ICU/IMV utilization among adults with CKD does not fully explain their excess of case fatality.

CKD significantly heightens the risk and severity of COVID-19 through several pathophysiological mechanisms.^36,40^ CKD induces chronic immune dysfunction, including impaired innate and adaptive immunity, loss of immunoglobulins, and reduced vaccine efficacy, resulting in higher viral loads and weaker protection against severe acute respiratory syndrome coronavirus 2, even postvaccination^36,37,41^ Furthermore, CKD promotes a proinflammatory and prothrombotic state that exacerbates COVID-19's complications, such as cytokine storms and coagulopathy.^37,42,43^ CKD also increases susceptibility to AKI during infection, a complication strongly associated with higher case fatality.^44,45^ These factors converge to create a vicious cycle, rendering COVID-19 particularly dangerous for patients with CKD and underscoring the necessity for tailored protective and therapeutic strategies in this vulnerable population.^36,37,41^

This study has several limitations. First, its retrospective design and reliance on a national surveillance database may introduce selection and misclassification biases. CKD was captured as a single binary variable, precluding evaluation by stage or kidney replacement modality; thus, the CKD group represents a heterogeneous mix likely enriched for advanced CKD, dialysis, and transplant recipients, while milder, unrecognized CKD in the comparison group is also probable. Such nondifferential misclassification would generally bias effect estimates toward the null. Comorbidities and outcomes were abstracted from administrative records and may be incomplete or inaccurate. Key clinical variables—laboratory data, treatments (including immunosuppression and antivirals), vaccination status, and cause of death—were unavailable, limiting adjustment and potentially leaving residual confounding, this is an intrinsic structural limitation of the nationwide surveillance system, not a design choice of the study. Place of death is not recorded, so we report case fatality among notified cases rather than setting-specific mortality. Although we incorporated municipal-level socioeconomic measures in sensitivity analyses, unmeasured determinants of access and quality of care may persist. As an observational study, the analyses support associations rather than causation. Finally, generalizability to settings with different health care systems or CKD care models may be limited.

Future studies should examine gradients of risk across CKD stages, dialysis types, and transplant status. They should also assess vaccine type, booster response, and hybrid immunity, including serological and cellular correlates of protection. Linkage of surveillance to clinical and laboratory data would improve adjustment and allow exploration of effect modification. Research on access barriers, triage practices, and treatment patterns could clarify mechanisms underlying excess risk. Quasi-experimental and implementation studies could test strategies such as prioritized boosting, early antiviral access, and equity-focused interventions in deprived settings.

In summary, in this nationwide analysis, CKD remained independently associated with higher case fatality and hospitalization across pandemic phases, with attenuation during 2022 followed by a resurgence of risk in 2024. Patients with CKD experienced disproportionately high fatality rates despite overall improvements in prevention and care, underscoring ongoing vulnerability. These findings support prioritized booster programs, rapid access to antivirals, and equity-oriented policies to strengthen care for people with CKD, alongside continued surveillance to detect shifts in risk as epidemiology and immunity evolve. Recognizing CKD as a critical comorbidity is essential for reducing health inequities and improving outcomes in this growing, high-risk population.

## Data Availability

All original data, including deidentified patient-level data or individual laboratory data measurements, are included in the manuscript and/or supplemental material.

## Declarative Statements

This study includes clinical experimentation and received Institutional Review Board or Ethics Committee approval. The need to obtain informed patient consent was waived. This study includes clinical experimentation and complies with the Declaration of Helsinki.

## Supplemental Material

Supplemental Table 1. STROBE checklist for cohort studies and locations of each reporting item within the manuscript.

Supplemental Table 2. Sociodemographic profile, comorbidities, and clinical outcomes of patients with laboratory-confirmed COVID-19 stratified by sex (January 2020–June 2025).

Supplemental Table 3. Descriptive characteristics and aORs for ICU admission and IMV in patients with laboratory-confirmed COVID-19.

Supplemental Table 4. Descriptive characteristics and aORs for case fatality by age group in patients with laboratory-confirmed COVID-19.

Supplemental Table 5. Descriptive characteristics and aORs for hospitalization by age group in patients with laboratory-confirmed COVID-19.

Supplemental Table 6. Descriptive characteristics and aORs for ICU admission by age group in patients with laboratory-confirmed COVID-19.

Supplemental Table 7. Descriptive characteristics and aORs for IMV by age group in patients with laboratory-confirmed COVID-19.

Supplemental Table 8. Sensitivity analysis restricted to ICU admissions: in-ICU case fatality by CKD status, overall and by age group.

Supplemental Table 9. Yearly aORs for CKD and other risk factors on COVID-19 case fatality (2020–2025).

Supplemental Table 10. National COVID-19 vaccination coverage in adults (≥18 years), Mexico 2020–2025.
